# Solvent effect modelling of isocyanuric products synthesis by chemometric methods

**DOI:** 10.1155/S1463924602000159

**Published:** 2002

**Authors:** Jean-Louis Havet, Myriam Billiau-Loreau, Catherine Porte, Alain Delacroix

**Affiliations:** Laboratoire de Chimie Industrielle et Génie des Procédés Conservatoire National des Arts et Métiers 2 rue Conté Paris F-75003 France

## Abstract

Chemometric tools were used to generate the modelling of solvent e¡ects on the N-alkylation of an isocyanuric acid salt. The method proceeded from a central composite design applied on the Carlson solvent classification using principal components analysis. The selectivity of the reaction was studied from the production of different substituted isocyanuric derivatives. Response graphs were obtained for each compound and used to devise a strategy for solvent selection. The prediction models were validated and used to search for the best selectivity for the reaction system. The solvent most often selected as the best for the reaction is the N,N-dimethylformamide.


Journal of Automated Methods & Management in Chemistry
Vol. 24, No. 4 (July–August 2002) pp. 111–119

### Solvent effect modelling of isocyanuric products synthesis by chemometric methods

**Jean-Louis Havet, Myriam Billiau-Loreau, Catherine Porte* and Alain Delacroix**
*Laboratoire de Chimie Industrielle et Génie des Procédés, Conservatoire National des Arts et Métiers, 2 rue Conté, F-75003 Paris, France*

*Chemometric tools were used to generate the modelling of solvent effects on the N-alkylation of an isocyanuric acid salt. The method proceeded from a central composite design applied on the Carlson solvent classification using principal components analysis. The selectivity of the reaction was studied from the production of different substituted isocyanuric derivatives. Response graphs were obtained for each compound and used to devise a strategy for solvent selection. The prediction models were validated and used to search for the best selectivity for the reaction system. The solvent most often selected as the best for the reaction is the N,N-dimethylformamide.*

### Introduction

The nature of solvents plays a leading part in the orientation of reaction products [1, 2]. To ensure the success of a planned reaction, a chemist must take the greatest care in the choice of solvents. The effects of solvents on chemical reactivity have been studied since the end of the nineteenth century, and their influence is generally approached in two ways: qualitatively and quantitatively. Qualitatively, the chemist uses the classification of solvents according to whether they are polar or apolar, protic or aprotic. The acid–base behaviour and the electron pair donor–acceptor effect are also considered. For a quantitative study, classifications are used that consider such physical constants as dipolar moment, dielectric constant, boiling point, etc. All these classifications are very useful when choosing from a wide range of common solvents. However, qualitative characteristics and physical parameters are usually inadequate to predict solvent influence correctly. Moreover, and unfortunately, not all classifications are homogeneous. For example, the classification for arranging solvents by growing order of dielectric constants is not the same as the dipolar moment classification. Therefore, a description of solvent effects through several parameters becomes very complicated, even problematical.

The most important criterion for solvent classification is 'polarity' [1]. The polarity of a solvent is generally used to characterize the set of interactions with the solute. However, this notion of polarity is difficult to define and quantify precisely. Polarity can be described quantitatively by physical parameters (dipolar moment, dielectric constant) or by empirical parameters (Dimroth–Reichardt constant, Kosower constant). These data can be considered as good but partial indicators of polarity. Furthermore, the accumulation of empirical values shows that none of these definitions is totally convenient. The use of several parameters that are not independent in multiparametric equations allows one to improve the quantification of polarity, but it remains insufficient to have a general classification of solvents [3].

The most ambitious approach for a general classification of solvents uses multivariate statistical methods [4, 5]. The compilation of the physicochemical constants of solvents allows one to take different properties simultaneously into account. At the end of the analysis, solvents can be regrouped into different classes where the properties and behaviour similarities and the correlatively dissimilarities appear. Moreover, the interest of the method lies in the geometric representation of solvents. Many statistical studies have been applied, helping to set up a few general classifications of solvents. Gramatica *et al.* [6] used different sets of molecular descriptors to make a general classification of 152 organic solvents. This work was developed from chemometric methods like the *k*-nearest neighbour. Chastrette [7] and Chastrette and Carretto [8] presented one of the first classifications made by principal components analysis (PCA). At the end of the analysis, 22 solvents described by six descriptors were ordered in different classes. Carlson [9], Carlson and Lundstedt [10] and Carlson *et al.* [11, 12] established many classifications that find different applications. They were constructed by PCA and were used, for instance, to study the influence of different solvents on the optimum reaction conditions.

The aim of this study was to predict, quantitatively, the selectivity of a *N*-alkylation of isocyanuric acid salt where the nature of the solvent exerts a considerable influence on different product ratio. General solvent classifications are also a fundamental necessity to allow for the elaboration of a model of solvent effects on the reaction. The determination of substituted product yields according to medium nature can improve the optimum conditions for solvent selection.

### Preliminary presentations

### *Reaction presentation*

The reaction studied is the *N*-alkylation of isocyanuric acid salt by nucleophilic substitution. The reaction presents a fundamental problem related to the nucleophilicity and basicity of the intermediate salt. The nucleophilic power normally leads to the mono-alkylated

* To whom correspondence should be addressed.

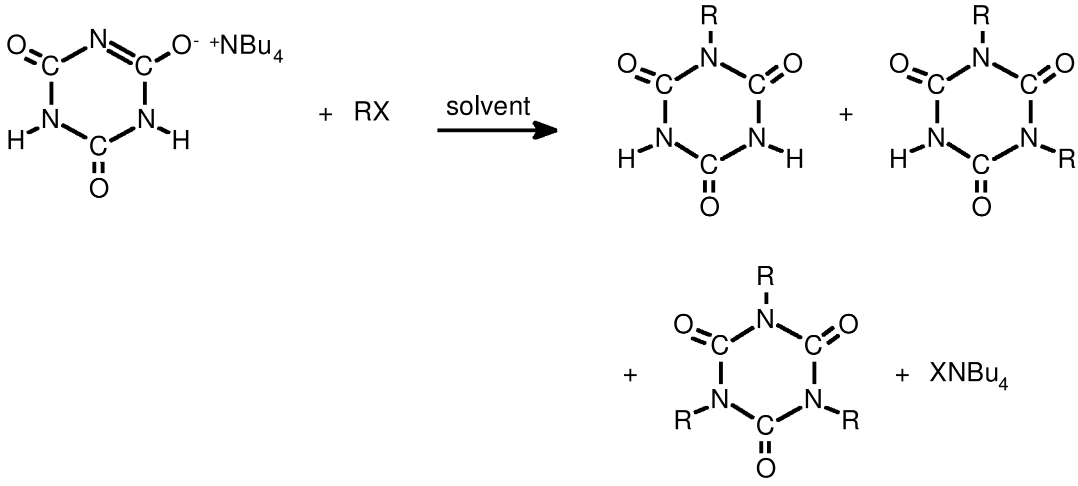

*Figure 1. Scheme 1.*

product, but the basic effect leads to polysubstitution on the heterocyclic compound, which explains why the synthesis has no selectivity. The reaction is presented in figure 1.

The isocyanurates used are usually alkaline and the substitution often takes place in aqueous phase. In this study, a new organic salt was synthesized, i.e. tetrabutylammonium isocyanurate. The nature of this salt allows one to have different levels of reactivity according to the medium. Tetrabutylammonium isocyanurate offers such interesting properties as solubility in organic solvents. Its reactivity in the organic phase was studied in order to influence the alkylation selectivity. The medium effects observed on the reaction are different depending on whether solvents are apolar or polar, for instance. The influence of protic solvents was relatively expected and easily explainable, but the conversion rate of the alkylation and the yields of the different substituted isocyanuric derivatives were impossible to estimate. The importance of the nature of the solvent where isocyanuric acid is involved had already been shown by Richard *et al.* [13]. Mono- or dihydroxymethyl isocyanuric products were synthesized by using one equivalent of isocyanuric acid with one or two equivalents of formaldehyde in pyridine. The yields of these reactions were about 90%. However, no product was formed when the same reaction took place in dimethylformamide or acetonitrile. These three aprotic polar solvents led to very different results. This can be explained by the difference in basicity. Screening solvents requires a suitable general classification.

### *Presentation of the Carlson classification* [9]

The solvent classification selected for our study comes from PCA made by Carlson. PCA is a multivariate statistical method that plays an increasingly important part in the field of chemometrics [14–16]. This statistical description leads to us have approximate, as well as optimal, graphic representations of table data. The user of the method will accept a loss in information to obtain a better legibility of the data [17]. PCA applies to a rectangular table where $n$ individuals are described by $p$ variables. Analysis leads to a minimal number of components that can be kept and thus determines a plane spanned by two (or three) principal components.

Carlson's solvent classification is one of the most general recorded so far. PCA was realized on the table data concerning the 103 common solvents used in organic chemistry characterized by nine descriptors. Apolar, polar aprotic and protic solvents appear in Carlson's list.

The nine descriptors used by Carlson are: melting point (°C); boiling point (°C); dielectric constant; dipolar moment ($D$); refractive index; normalized Reichardt–Dimroth constant; density; lipophilicity (the logarithm of the equilibrium constant of the distribution of the solvent between 1-octanol and water at 25 °C); and water solubility (logarithm, mol $l^{-1}$).

The multivariate analysis of the 103 solvents characterized by nine descriptors leads to the determination of the first two principal components where each solvent has scores. Table 1 reproduces an extract of Carlson's PCA study. From the scores $t_1$ and $t_2$, the Carlson map of 103 solvents can be represented in the first two components (figure 2).

Statistical analysis allows one to measure the influence of variables on principal components. Thus, the Carlson map can be interpreted in terms of polarity and polarizability. Indeed, the first component is strongly correlated with such typical descriptors of polarity as dipolar moment, dielectric constant or the Dimroth–Reichardt constant, whereas the second one is mainly described by the refractive index and the density. The properties of 'polarity' and 'polarizability' brought out from the solvent analysis seem particularly suitable for the study of such a reactional system as bimolecular substitution.

### *Experimental*

The isocyanurate derivatives were synthesized according to the following steps. For example, tetrabutylammonium isocyanurate (0.022 moles) was stirred in 100 ml of the solvent studied at 25 °C. Methyliodide (0.026 moles) was introduced. The reactional mixture was

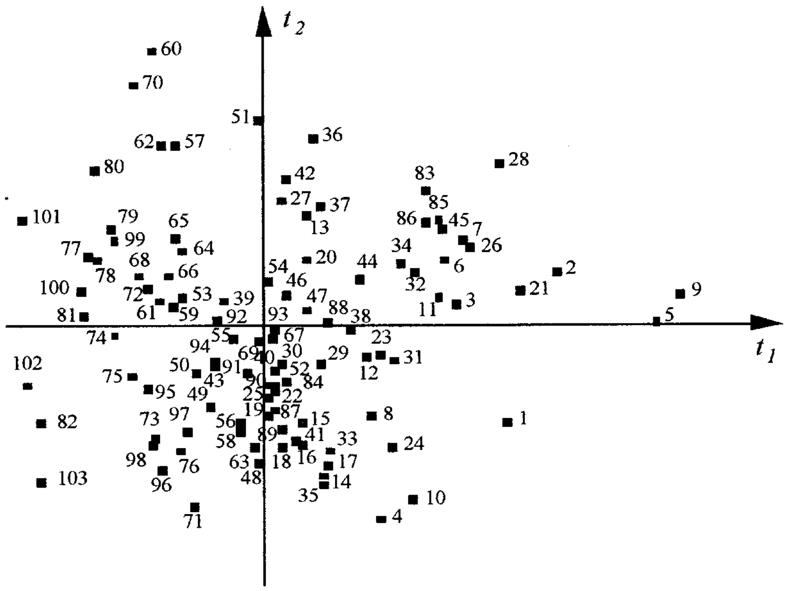

| | | | | | |
|---|---|---|---|---|---|
| 1 | Water | 42 | Acetophenone | 94 | Diethylcarbonate |
| 2 | Formamide | 43 | Dichloromethane | 95 | 1,1-Dichloroethene |
| 3 | 1,2-Ethanediol | 44 | 1,1,3,3-Tetramethylurea | 96 | *tert*-Butylmethylether |
| 4 | Methanol | 45 | HMPT | 97 | Diethylamine |
| 5 | *N*-Methylformamide | 46 | Cyclohexanone | 98 | Dipropylether |
| 6 | Diethylene glycol | 47 | Pyridine | 99 | 1,4-Dimethylbenzene |
| 7 | Triethylene glycol | 48 | Methylacetate | 100 | Tributylamine |
| 8 | 2-Methoxyethanol | 49 | 4-Methyl-2-pentanone | 101 | *cis*-Decaline |
| 9 | *N*-Methylacetamide | 50 | 1,1-Dichloroethane | 102 | Heptane |
| 10 | Ethanol | 51 | Quinoline | 103 | Pentane |
| | .../... | | .../... | | |

*Figure 2. Carlson map of the 103 solvents [9].*

*Table 1. Extract of the Carlson PCA study [9].*

| Solvent | Descriptors | | | | | | | | | Scores | |
|---|---|---|---|---|---|---|---|---|---|---|---|
| | 1 | 2 | 3 | 4 | 5 | 6 | 7 | 8 | 9 | $t_1$ | $t_2$ |
| 1. Water | 0.00 | 100.00 | 78.30 | 5.9 | 1.3330 | 1.000 | 0.9282 | −1.38 | 1.774 | 3.70 | −1.48 |
| 2. Formamide | 2.55 | 210.50 | 111.00 | 11.2 | 1.4475 | 0.799 | 1.1334 | −1.51 | 1.401 | 4.47 | 0.86 |
| 3. 1,2-Ethanediol | −12.60 | 197.15 | 37.70 | 7.7 | 1.4318 | 0.790 | 1.1088 | −1.36 | 1.060 | 2.90 | 0.34 |
| 4. Methanol | −97.70 | 64.50 | 32.66 | 5.5 | 1.3284 | 0.762 | 0.7914 | −0.77 | 1.393 | 1.76 | −2.98 |
| ⋮ | | | | | | | | | | | |
| 102. Heptane | −90.60 | 98.40 | 1.92 | 0.0 | 1.3876 | 0.012 | 0.6838 | 4.57 | −4.046 | −3.71 | −0.92 |
| 103. Pentane | −129.7 | 36.10 | 1.84 | 0.0 | 1.3575 | 0.009 | 0.6262 | 3.39 | −3.129 | −3.48 | −2.38 |

(1) Melting point (°C); (2) boiling point (°C); (3) dielectric constant; (4) dipolar moment (D); (5) refractive index; (6) normalized Reichardt–Dimroth constant; (7) density; (8) lipophilicity (log); (9) water solubility (log, mol $l^{-1}$).

stirred for 36 h at 25 °C to prepare methylisocyanurate and its derivatives di- and trialkylated.

Depending on the nature of the solvent, the mixture was heterogeneous or homogeneous. The separation of an heterogeneous mixture was performed by centrifugation. The solid phase was filtered off, then washed twice with 40 ml dichloromethane. The solid was separated by a new centrifugation. The dichloromethane phases were collected, then the solvent was removed by evaporation under vacuum. A second solid was recovered. Lastly, the reactional solvent was also removed by evaporation under vacuum to recover a solid phase, which was dried out. All the solid parts were analysed by HPLC to quantify the isocyanuric derivatives.

### Solvent effects modelling

The work carried out on the general classification of solvents made by Carlson then allowed a modelling of the reaction of the isocyanurate ammonium salt on the alkyl halogenate. The solvents used were actually defined by two scores characterizing the physicochemical properties of the medium. A function like yield variation can be

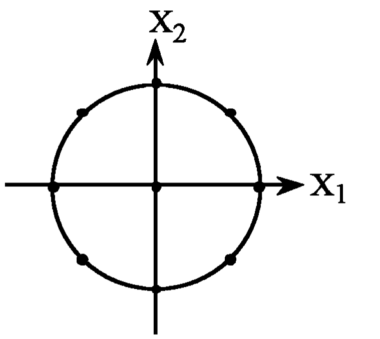

*Figure 3. Distribution of the central composite design points for two variables.*

observed in the function of these two scores. The modelling principle selected is turned towards the use of experimental designs to obtain the representative response function.

### *Model elaboration*

#### *Design choice*

In a first stage, some solvents in the Carlson map have to be found matching points in an experimental design from which a second-degree model can be derived. This design is the most suitable to represent a response surface. This kind of second-degree polynomial with two variables is described by equation (1):

$$Y = b_0 + b_1X_1 + b_2X_2 + b_{11}X_1^2 + b_{22}X_2^2 + b_{12}X_1X_2 \quad (1)$$

The most widely distributed experimental designs enabling one to obtain these polynomial parameters are central composite designs consisting of $N_f$ factorial points, $N_a$ axial points and $N_0$ centre points. For this study, $N_0 = 5$ was chosen so as to have a design with uniform precision [18–20]. The distribution of central composite design points for two variables is shown in figure 3.

$Y$ is the function of a yield product; $X_1$ and $X_2$ are the variables representing the medium nature. These variables take the scores of the principal components obtained in Carlson's analysis.

#### *Experimental point search*

The application of an experimental design may be made in several ways on the Carlson map. We can, for instance, try to reproduce the distribution of design points as accurately as possible on the map. However, this is very difficult if not impossible task. An inverse step was taken up to define the design points. The central composite design in fact was developed from the different experiments made. Presented is the experimental phase that led to the definition of the axial and factorial points. The centre point was only determined afterwards.

First, solvents with boiling points >160 °C were excluded from the experiment. Thirty-four solvents on the Carlson map, most placed in the higher part of the plane ($t_2 > 0$), were eliminated. This operative constraint obliges one to work in a reduced space of the plane.

From the solvents tested for the reaction and their situations on the map, an ellipse was elaborated. Its construction had to satisfy two conditions: first, to be sufficiently important to count a sizeable number of solvents, and, second, to cover different types of solvents (protic, polar aprotic, apolar).

In fact, all the justification for using the map and model rests in the capacity of one to provide the reaction selectivity for all kinds of solvents.

The circumference of the ellipse selected corresponds to the points defined by the following solvents: DMF, acetonitrile, acetone, diethylether, pentane, heptane, cyclohexane and chloroform. The figure centre was determined geometrically and corresponded to the point defined by solvent: 4-methyl-2-pentanone. Figure 4 shows the distribution of the set points on the Carlson map.

In the same way, the ellipse is defined by three aprotic polar solvents (DMF, acetonitrile, acetone) whose behaviours are very different, three apolar solvents (alcanes) and three aprotic solvents, which are slightly polar. Protic solvents are covered by the geometrical figure and can be used as validation points for the model.

The choice of an ellipse for the experimental design construction was made on account of it being easy to

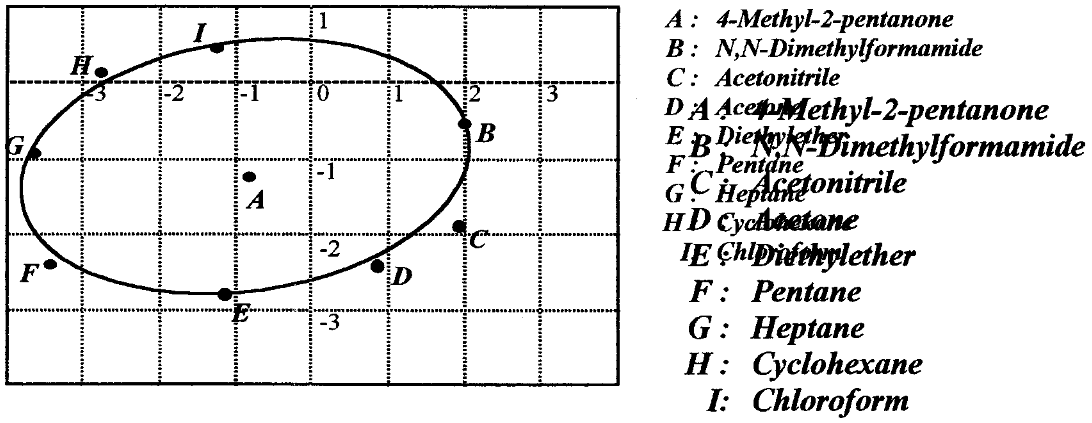

*Figure 4. Solvents' choice for modelling.*

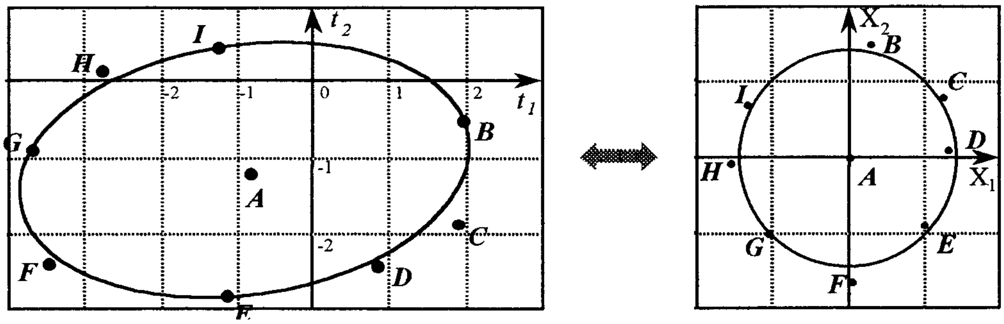

*Figure 5. Transformation of the ellipse into a central composite design.*

*Table 2. Transformation of the principal components into the normalized coordinates of central composite design.*

| Solvent | Principal components | | Centred reduced | | Matrix coordinates | |
|---|---|---|---|---|---|---|
| | $t_1$ | $t_2$ | $X_1'$ | $X_2'$ | $X_1$ | $X_2$ |
| DMF | 1.98 | −0.53 | 1.38 | −0.55 | 0.291 | 1.508 |
| Acetonitrile | 1.93 | −1.88 | 1.40 | 0.63 | 1.239 | 0.813 |
| Acetone | 0.85 | −2.44 | 0.84 | −1.04 | 1.33 | 0.094 |
| Diethylether | −1.13 | −2.80 | −0.21 | 1.48 | 1.022 | −0.89 |
| Pentane | −3.48 | −2.38 | −0.14 | −1.35 | 0.053 | −1.64 |
| Heptane | −3.70 | −0.92 | −0.96 | 1.23 | −1.05 | −1 |
| Cyclohexane | −2.78 | 0.16 | −1.43 | 0.29 | −1.56 | −0.090 |
| Chloroform | −1.26 | 0.45 | −1.31 | −0.98 | −1.33 | 0.678 |
| 4-Methyl-2-pentanone | −0.84 | −1.25 | 0 | 0 | 0 | 0 |

elaborate and transform into a circle by mathematical operators.

The solvents describing the ellipse can be selected only if the scores of the principal components can be transformed into the coordinates of the central composite design. The coordinates on the map of the nine solvents are first centred and reduced on the point representing 4-methyl-2-pentanone. A rotation of 55° is then executed on the intermediate values to obtain coordinates as near to the design points as possible. Table 2 gives the solvent scores and their transformations; figure 5 schematizes the transformation of the Carlson map ellipse into a central composite design with two variables.

The details of the calculation were as follows.

- Principal components scores of solvents were first centred and reduced:

$$X_1' = \frac{t_1(\text{compound } i) - t_1(\text{4-methyl-2-pentanone})}{C_1},$$

where

$$C_1 = \frac{\max(t_1) - \min(t_1)}{2\sqrt{2}};$$

$$X_2' = \frac{t_2(\text{compound } i) - t_2(\text{4-methyl-2-pentanone})}{C_2},$$

where

$$C_2 = \frac{\max(t_2) - \min(t_2)}{2\sqrt{2}}.$$

- The coordinates $X_i'$ were then changed by a rotation of 55° to obtain the coordinates $X_i$, which were used for the experimental design:

$$X_1 = X_1'(\text{compound } i) \times \cos(55 \times \pi/180) - X_2'(\text{compound } i) \times \sin(55 \times \pi/180)$$

$$X_2 = X_1'(\text{compound } i) \times \sin(55 \times \pi/180) + X_2'(\text{compound } i) \times \cos(55 \times \pi/180)$$

The calculation of the matrix from the composite design points allows one to validate the normalized coordinates. Nevertheless, figure 5 shows that they present a difference from the ideal (cf. figure 3).

### *Study of central composite design*

The responses selected are the yields of mono-, di- and trisubstituted isocyanuric products. The calculations of coefficients are realized from the matrix coordinates and experimentation results (table 3). All the operations executed on the matrix and calculations between matrices are realized by Excel® (Microsoft).

Polynomial coefficients $b_i$ are determined by:

$$[b] = [(X^t.X)^{-1}.X^t].[Y_{\text{exp}}],$$

where $Y_{\text{exp}}$ is the response matrix, which can be $Y_{\text{mono}}$, $Y_{\text{di}}$ and $Y_{\text{tri}}$.

*Table 3. Data of the central composite design.*

| Solvent | Matrix coordinates | | Yield (%) | | |
|---|---|---|---|---|---|
| | $X_1$ | $X_2$ | Mono- | Di- | Tri- |
| DMF | 0.291 | 1.508 | 35.6 | 14.7 | 6.1 |
| Acetonitrile | 1.239 | 0.813 | 17.0 | 11.8 | 14.2 |
| Acetone | 1.33 | 0.094 | 15.4 | 9.6 | 15.6 |
| Diethylether | 1.022 | −0.89 | 21.8 | 7.5 | 4.2 |
| Pentane | 0.053 | −1.64 | 23.4 | 11.8 | 5.4 |
| Heptane | −1.05 | −1 | 23.0 | 11.5 | 5.1 |
| Cyclohexane | −1.56 | −0.09 | 15.8 | 12.1 | 4.6 |
| Chloroforme | −1.33 | 0.678 | 17.8 | 8.8 | 6.8 |
| 4-Methyl-2-pentanone | 0 | 0 | 22.3 | 14.4 | 7.8 |
| 4-Methyl-2-pentanone | 0 | 0 | 22.0 | 12.5 | 7.0 |
| 4-Methyl-2-pentanone | 0 | 0 | 22.3 | 13.9 | 8.8 |
| 4-Methyl-2-pentanone | 0 | 0 | 24.0 | 14.9 | 7.9 |
| 4-Methyl-2-pentanone | 0 | 0 | 20.7 | 13.9 | 8.5 |

The three following models were established:

- Model of monosubstituted product yield:

$$Y_{mono} = 22.5 - 0.9X_1 + 2.4X_2 - 3.6X_1^2 + 3.1X_2^2 + 0.2X_1X_2.$$

- Model of disubstituted product yield:

$$Y_{di} = 13.8 - 0.8X_1 + 0.9X_2 - 2X_1^2 - 0.5X_2^2 + 1.9X_1X_2.$$

- Model of trisubstituted product yield:

$$Y_{tri} = 7.9 + 2.5X_1 + 0.8X_2 + 0.9X_1^2 - 1.3X_2^2 + 1.7X_1X_2.$$

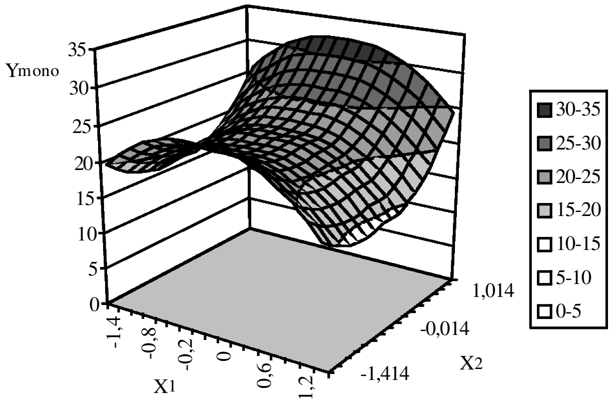

*Figure 6. Representation of the prediction model $Y_{mono}$.*

### Results and discussion

The halogenate/isocyanuric acid salt ratio must be always 1.2 if preparation of the monosubstituted product is preferred. The experimental process is very long so that the system cannot evolve longer. Thus, for each test, the distribution of substituted products can be observed comparatively and the reaction can be compared selectivity only in relation to solvent nature. Therefore, the interest in this work is to set up a method for solvent selection by chemometric tools.

### *Graphical representation of models*

After the necessary statistical verifications were made (SD analysis, residues analysis), the three response models appeared to be satisfactory for an estimation study.

A representation of the three response surfaces (figures 6–8) was drawn in the design field in order to observe the potential presence of an optimum or to follow the yield evolution more easily.

The response surface of monosubstituted isocyanuric product yield was typically saddle-like in shape (figure 6). The point associated to DMF corresponded to the optimum of the surface. Observation of the surface of $Y_{di}$ (figure 7) immediately showed the reaction selectivity as being less satisfactory because an optimum was present in the same area. $Y_{tri}$, whose axes $X_1$ and $X_2$ are reversed in their representation (figure 8), is also a saddle-shaped, but a deformed one.

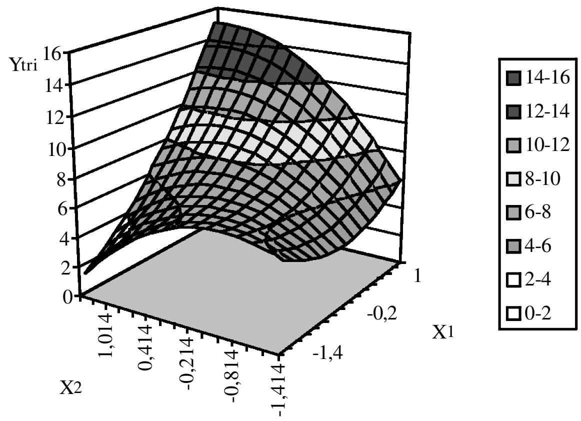

*Figure 7. Representation of the prediction model $Y_{di}$.*

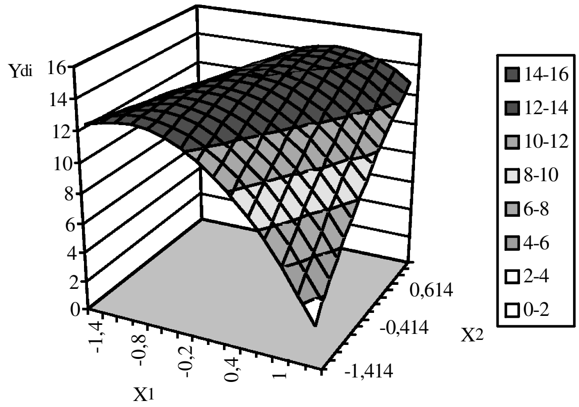

*Figure 8. Representation of the prediction model $Y_{\text{tri}}$.*

*Experimental validation*

To confirm model validity, the results of a few experimental tests carried out with other solvents were compared with the responses calculated with established polynomials. The solvents selected must necessarily be in the study field and must, if possible, be of a different nature.

The validation solvents are: toluene, diisopropylether and 2-propanol. Figure 9 shows the three solvents on the design plane. Toluene is aprotic apolar, diisopropylether is slightly polar aprotic and propanol is protic. These solvents are used for the same model and the choice of validation points is of interest because two of them are on axis $X_1$ and one on axis $X_2$. Unfortunately, no point representing a solvent located in the centre of a quarter has been found.

Table 4 gives all the results for the experimental and calculated yields for the three models. Several intervals exist surrounding the experimental values. The interval selected is *prediction interval*, defined by equation (2):

$$\hat{Y}_A - t \cdot \sigma\sqrt{1 + [X_A]^t \cdot [X^t \cdot X]^{-1} \cdot [X_A]} < Y_A < \hat{Y}_A + t \cdot \sigma\sqrt{1 + [X_A]^t \cdot [X^t \cdot X]^{-1} \cdot [X_A]}, \quad (2)$$

where

- $Y_A$ is the experimental value for the point A,
- $\hat{Y}_A$ is the calculated value for the point A,
- $t$ is the Student's $t$ parameter for $N - p$ degrees of freedom and risk $\alpha$ ($t = 2, 3$),
- $\sigma$ is the SD for point A,
- $[X_A]$ is the model matrix for point A.

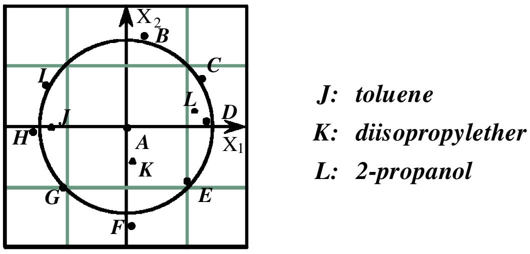

*Figure 9. Situation of the three solvents for the validation.*

According to the criteria of uniform precision, for each model the deviation of the calculated yield is nearly constant. This is expected because the design is quite elaborate.

In table 4, all the experimental yields are included correctly in the evaluated intervals around the calculated values. Therefore, the models can be considered as valid.

If the SDs are studied more precisely, a slight lack of adjustment in the $Y_{\text{mono}}$ and $Y_{\text{tri}}$ prediction models is observed, although the experimental error is very weak. Centred points allow one to calculate an estimation of the experimental error, and axial and factorial points allow one to determine the lack of adjustment for each model (table 5). This can be easily explained because a few points of the matrix are slightly off-centre compared with the theoretical coordinates (e.g. points C and I in figure 5). Therefore, the elaborated models present some distortions. According to the definition, there is also a lack of precision for the interval.

This lack of precision can be due to several reasons. First, experimentation is a source of errors. Second, design elaboration from off-centre points can explain a divergence between real and estimated values. Moreover, the Carlson map can be improved in relation to the reaction system by including more appropriate parameters to the reactional system studied such as donor number (DN) or acceptor number (AN), so as to obtain more significant principal components of synthesis criteria. The solvents will have to be more precisely defined, with new scores that are more efficient in relation to the nucleophilic substitution. This new principal components analysis, which is very easy realized by specialized software, would give theoretical models of solvent maps more in keeping with experimental results.

The superimposition of the three isocontour plots (figure 10) shows that ideal selectivity, i.e. maximal monosubstituted product yield and minimal di- and trisubstituted products yields, does not satisfactorily appear in the field

*Table 4. Models validation.*

| | ACP scores | | Expermental matrix | | Yield mono- (%) | | Yield di- (%) | | Yield tri- (%) | |
|---|---|---|---|---|---|---|---|---|---|---|
| Solvent | $t_1$ | $t_2$ | $X_1$ | $X_2$ | Experimental | Calculated | Experimental | Calculated | Experimental | Calculated |
| Toluene | −2.30 | −0.11 | −1.23 | −0.03 | 23.3 | 17.9 ± 7.8 | 11.2 | 12.1 ± 3.3 | 4.9 | 6.2 ± 6.2 |
| Diisopropylether | −1.69 | −1.73 | 0.10 | −0.59 | 21.0 | 22.2 ± 7.6 | 9.8 | 13.0 ± 3.2 | 6.1 | 7.2 ± 6.0 |
| 2-Propanol | 0.90 | −2.16 | 1.15 | 0.25 | 15.0 | 17.4 ± 7.9 | 11.8 | 10.8 ± 3.4 | 10.0 | 12.6 ± 6.3 |

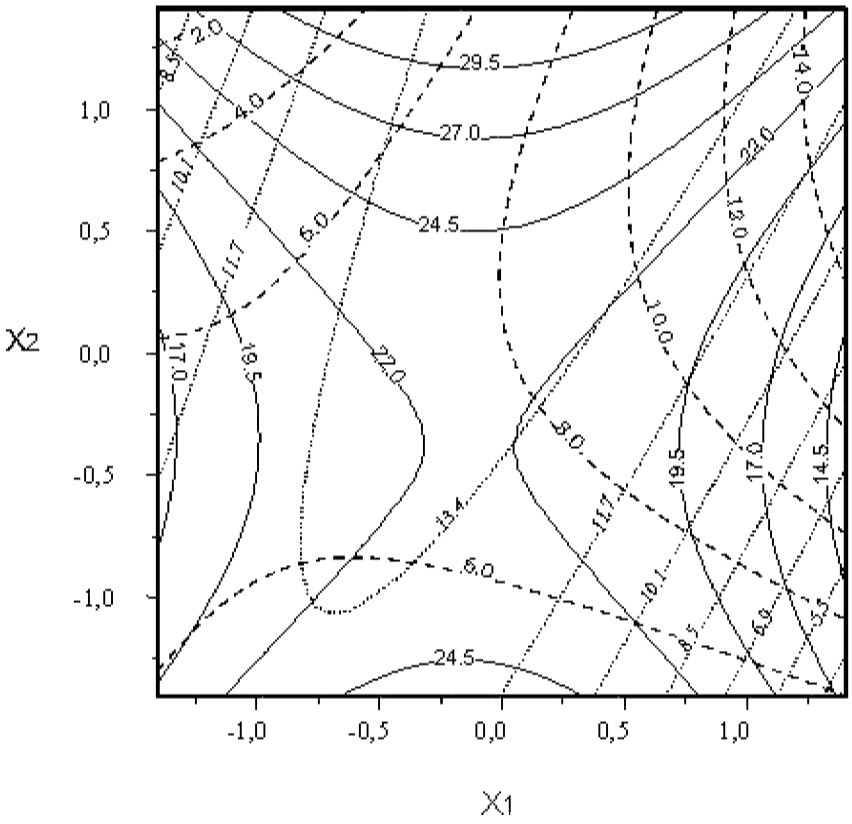

*Figure 10. Isocontour plots for the models. ——, Mono-; · · · · · ·, Di; - - - - -, trisubstituted products.*

*Table 5. Experimental error and lack of adjustment.*

| SD | Mono- | Di- | Tri- |
|---|---|---|---|
| Experimental error | 1.2 | 0.9 | 0.7 |
| Lack of adjustment | 4.4 | 1.6 | 3.6 |

studied nor does it for the experimental conditions. Indeed, mono- and disubstituted products yields are maximal in the same area.

In terms of principal components, the best solvent is the compound that is the most strongly positively correlated with the first component and the least correlated with the second. In figure 4, DMF (solvent B) is that solvent. It gives the most important monomethyl isocyanurate yield and one of the best reaction selectivities. The polar aprotic solvent behaviour was expected because this kind of solvent nature is particularly appropriate for bimolecular substitution. What is more interesting is the good selectivity in diethylether, although the yields are not so high. This is a compound that is only strongly correlated with the polarizability component.

### Conclusions

A new approach of solvent effect quantification has been proposed. Starting from a general classification of solvents and from experimental design, models have been established enabling one to predict reaction yields quite reliably. In a first step, the PCA made by Carlson on 103 solvents described by nine descriptors was used because statistical analysis allowed one to draw up an appropriate solvent map taking into account many properties. From this map, a central composite design was elaborated to draw response surfaces of product yields in relation to solvent nature.

The new method applied here is simple and allows one to select the best solvent for a reactional system. It has been tested for a special reaction, but it can now be developed for many other reactional systems.

Although models lack precision, overall they are satisfactory and can be considered as valid because experimental and estimated values match. The solvent classification has to be selected in relation to the chemical system. Principal components analysis can be improved, for example, by adding a choice of sensitive descriptors.

Chemometric tools are very powerful means to estimate or model an effect. Nevertheless, in chemistry, estimations of optimum conditions always have to be verified experimentally.

### Acknowledgements

The authors thank M. Henri FAUDUET for his help.

### References

1. Reichardt, C., *Solvents and Solvent Effects in Organic Chemistry*, 2nd edn (Weinheim: VCH, 1990).
2. Reichardt, C., *Pure Appl. Chem.*, 54 (1982), 1867.

3. Reichardt, C., *Angew. Chem. Int. Ed. Engl.*, **18** (1979), 98.
4. Maria, P.-C., Gal, J.-F., de Franceschi, J. and Fargin, E., *J. Am. Chem. Soc.*, **109** (1987), 483.
5. Chastrette, M., Rajzmann, M., Chanon, M. and Purcell, K. F., *J. Am. Chem. Soc.*, **107** (1985), 1.
6. Gramatica, P., Navas, N. and Todeschini, R., *Trend. Anal. Chem.*, **18** (1999), 461.
7. Chastrette, M., *Tetrahedron*, **35** (1979), 1441.
8. Chastrette, M. and Carretto, J., *Tetrahedron*, **38** (1982), 1615.
9. Carlson, R., *Design and Optimization in Organic Synthesis* (Amsterdam: Elsevier, 1992).
10. Carlson, R., Lundstedt, T. and Shabana, R., *Acta Chem. Scand.* B, **40** (1986), 694.
11. Carlson, R. and Lundstedt, T., *Acta Chem. Scand.* B, **41** (1987), 164.
12. Carlson, R., Lundstedt, T. and Albano, C., *Acta Chem. Scand.* B, **39** (1985), 79.
13. Richard, B., Richard, M. and Lenzi, M., *Bull. Soc. Chim. Fr.*, **127** (1990), 461.
14. Cibois, P., *L'Analyse Factorielle* (Paris: PUF, 1994).
15. Bouroche, J.-M. and Saporta, G., *L'Analyse des Données* (Paris: PUF, 1998).
16. Dazy, F. and Le Barzic, J.-F., *L'Analyse des Données Évolutives, Méthodes et Applications* (Paris: Technip, 1996).
17. Morineau, A. and Aluja-Banet, T., *Analyse en Composantes Principales* (Saint-Mandé: CISIA-CERESTA, 1998).
18. Delacroix, A. and Porte, C., *Anal. Mag.*, **24** (1996), M22.
19. Delacroix, A. and Porte, C., *Techniques de l'ingénieur*, **P225** (1987), 1.
20. Goupy, J., *Plans d'Expériences pour Surfaces de Réponse* (Paris: Dunod, 1999).

